# Lies, irony, and contradiction — an annotation of semantic conflict in the movie "Forrest Gump"

**DOI:** 10.12688/f1000research.9635.1

**Published:** 2016-09-26

**Authors:** Michael Hanke, Pierre Ibe

**Affiliations:** 1 Psychoinformatics Lab, Department of Psychology, University of Magdeburg, Magdeburg, 39106, Germany; 2 Center for Behavioral Brain Sciences, Magdeburg, Germany

**Keywords:** semantic, cognition, brain imaging, frequency statistics

## Abstract

Here we extend the information on the structure of the core stimulus of the studyforrest project (http://studyforrest.org) with a description of semantic conflict in the “Forrest Gump” movie. Three observers annotated the movie independently regarding episodes with portrayal of lies, irony or sarcasm. We present frequency statistics, and inter-observer reliability measures that qualify and quantify semantic conflict in the stimulus. While the number of identified events is limited, this annotation nevertheless enriches the knowledge about the complex high-level structure of this stimulus, and can help to evaluate its utility for future studies, and the usability of the existing brain imaging data regarding this aspect of cognition.

## Introduction

Detection of semantic conflict is an important cognitive skill for human social interaction. It is required to identify lies (false statements made with the intention to deceive) but also to correctly interpret stylistic devices — such as sarcasm and irony (statements with direct meaning that is the opposite ^1^ or contrary ^2^ to the implied semantic content). As the interpretation of such events is highly context dependent, it is difficult to study how the brain processes these in the context of real-life like interactions in complex natural environments.

In this study we explored occurrences of semantic conflict in the core stimulus of the studyforrest project ( http://studyforrest.org) — the motion picture “Forrest Gump” — in order to evaluate whether the available brain imaging data ^3, 4^ can be readily used to study this aspect of cognition. We annotated the presence of contradictory statements, including lies and ironic statements, as well as the portrayal of cues, such as exaggeration or raised eyebrows, that are often associated with making ironic statements. Additionally, we recorded the context that allowed observers to classify an event as contradictory.

Depending on the exact criterion used for identifying events across observers, we found only between 64 and 36 occurrences of semantic conflict or portrayal of irony cues in the entire movie stimulus. These are likely insufficient numbers for an investigation based on these data alone. However, these new annotations nevertheless contribute to a more comprehensive description of this complex movie stimulus ^5, 6^ and may be useful as confound variables in subsequent studies.

## Materials and methods

### Stimulus

The annotated stimulus was a slightly shortened (≈2 h) version of the movie Forrest Gump (R. Zemeckis, Paramount Pictures, 1994), with a dubbed German soundtrack, and is identical to the audio-visual movie annotated in 5, 6. Further details on this particular movie cut, and how to reproduce it from commercially available sources, are available in 4.

### Observers

Three observers (all female, age 19–20) independently annotated the movie. They were also involved in the development of the concept for this annotation.

### Procedure

Observers were instructed to watch the movie from beginning to end, replaying scenes as often as required, and to detect two types of events: 1) whenever a verbal statement is made that contradicts with either the immediate context or with the viewer’s body-of-knowledge at this point in the movie, or 2) whenever one or more cues associated with irony (predefined list, see below) are portrayed. In either case, observers had to describe the event by specifying its properties via a number of variable settings in a spreadsheet. The software video player VLC ( http://www.videolan.org/vlc) was used to watch and navigate through the movie.

## Data legend

For each annotated event, a total of 10 properties were recorded, each of which are described in the following sections.

**Start and end** The duration of each event is recorded in start and end as the number of seconds from movie start (no sub-second precision, due to limitations of the video player time display). The time-points correspond to the onset and offset of the respective evidence. Both times can be identical in the case of events with less than one second duration. For contradictory statements, the duration covers the time from the onset of evidence of a contradiction until the end of the statement.

**Sender and receiver** The identity of a character making a contradictory statement or portraying an irony cue is encoded in sender using character labels listed in 5. In the case that the respective statement is directed to another present movie character, its identity is encoded in receiver.

**Evidence of a contradiction** The contradiction flag indicates the presence of a contradiction in an event (1: present, 0: absent). The variable proof qualifies if the current or previous events provide the viewer with information to allow the detection of this contradiction (see Table 1). If proof is empty, the movie itself does not contain such information (e.g. a common sense contradiction).

**Table 1.** Definitions for all annotation codes.

Code	Description
*Cues* RHETORICALQ UNDERSTATEMENT EXAGGERATION LAUGH RAISEDEYEBROW EYEROLLING TEMPOCHANGE TONECHANGE PAUSE	statement that is or contains a rhetorical question a fact is stated in a weakened manner a fact is overstated a statement accompanied by laughter character makes a statement while raising an eyebrow character makes a statement while rolling his/her eyes a change of the speech tempo within a sentence or compared to previous sentences a change of the tone, pitch, or volume within a sentence or compared to previous sentences an intentionally placed (yet still unexpected) or unnatural pause within a sentence
*Contradiction proof* IMMEDIATE PAST	a statement that contradicts with information in the immediate context a statement that contradicts with previous actions or information given in the past
*Conflict category* IRONY LIE	an ironic statement (a statement with a direct meaning that is the opposite or contrary to its implied semantic content) a lie (a false statement made with the intention to deceive)

**Irony cues** The variable cues contains a space-separated list of labels for all irony cues present in a particular events. See Table 1 for a description of all possible labels.

**Event category** The category variable classifies events into lies, ironic statements, and other events (value empty).

**Intention** Two more variables encode whether a contradiction was used deliberately and whether this was noticed by the receiver. The variable intended encodes the presence of evidence for deliberate use (1: yes, 0: no). The variable is empty if there is no evidence for either case. The second variable intention_decoded encodes, in the same way, whether a potential receiver noticed a deliberate ironic statement or lie.

### Dataset content

The released annotation are three, text-based, comma-separated-value (CSV) formatted tables ( data/o??.csv), one for each observer.

The source code for all descriptive statistics included in this paper is available in code/descriptive_stats.py (Python script).

Events of semantic conflict and occurrences of irony cues in the motion picture "Forrest Gump" Raw data are presented in csv tables. Click here for additional data file. Copyright: © 2016 Hanke M and Ibe P 2016 Data associated with the article are available under the terms of the Creative Commons Zero "No rights reserved" data waiver (CC0 1.0 Public domain dedication).

Python script The Python script to compute all descriptive statistics presented in the paper from the released annotations is provided. Click here for additional data file. Copyright: © 2016 Hanke M and Ibe P 2016 Data associated with the article are available under the terms of the Creative Commons Zero "No rights reserved" data waiver (CC0 1.0 Public domain dedication).

## Dataset validation

We used an automated procedure to check the annotation records of individual observers for errors or potential problems. Observers submitted their annotations in tabular form to a script that generated a list of error and warning messages. Using this feedback, observers double-checked their annotations as often as necessary until no objective errors were found and all warning messages were confirmed to be false positives. The tests included, for example, plausibility of timing information (no end time before the respective start time) or the presence of unknown condition labels.

In order to assess inter-observer agreement of annotations, we used a two-step approach. First, the temporal location of events depicting *any* relevant property were determined by comparing annotation timing across observers. The columns in Table 2 report agreement statistics for events defined by at least one, two, or all three observers recording an annotation for the same sender at the same time. In the case that individual observers reported events of different length, or with only partially overlapping duration, only the time-windows with the minimum number of observers reporting an event were considered.

**Table 2.** Annotation inter-observer agreement statistics. Number of events and categorization agreement are presented for three levels of inter-observer agreement on the temporal location and the performing movie character. The number of events for any particular event property are determined by majority vote across observers, i.e. an event is counted when more observers indicate the presence of a property than its absence. Exhaustive technical detail on the statistical analysis can be found in the descriptive_stats.py Python script.

	Event location min. agreement		
	33%	66%	100%
Number of events Mean event duration (s) Mean event distance (s)	64 6.4 80.6	46 5.4 100.5	36 4.6 131.6
*Event types* [majority vote counts and inter- observer agreement (Fleiss’ Kappa)]			
Contradiction Irony Lie	36 (0.79) 20 (0.90) 7 (0.72)	36 (0.84) 20 (0.91) 7 (0.76)	32 (1.00) 19 (0.93) 6 (0.85)
*Contradiction evidence* [majority vote counts and inter- observer agreement (Fleiss’ Kappa)]			
Immediate context Memory	34 (0.42) 11 (0.62)	35 (0.57) 11 (0.71)	28 (0.79) 10 (0.79)
*Cue type occurrences* [majority vote counts and inter- observer agreement (Fleiss’ Kappa)]			
Rhetorical question Understatement Exaggeration Laughter Raised eyebrow Eye rolling Tempo change Tone change Pause	13 (0.70) 2 (0.66) 6 (0.59) 8 (0.73) 13 (0.68) 2 (0.66) 1 (0.48) 14 (0.64) 4 (0.51)	13 (0.89) 2 (0.79) 6 (0.65) 8 (0.89) 14 (0.73) 2 (0.79) 1 (0.58) 14 (0.67) 4 (0.58)	11 (0.96) 2 (0.79) 6 (0.68) 7 (0.94) 11 (0.81) 1 (1.00) 1 (0.74) 12 (0.76) 4 (0.56)

In the second step, we computed Fleiss’ Kappa ^7^ for each individual property of an annotation separately with respect to being consistently assigned or non-assigned to the identified events ( Table 2). We observe increasing inter-observer agreement of all annotated properties with increasing agreement of annotation timing, approaching “substantial” or “almost perfect” agreement — according to the conventions put forth by 8.

## Data and software availability

F1000Research: Dataset 1. Events of semantic conflict and occurrences of irony cues in the motion picture "Forrest Gump", 10.5256/f1000research.9635.d136203 ^9^

F1000Research: Dataset 2. Python script, 10.5256/f1000research.9635.d136204 ^10^

In addition, released data, code, and manuscript sources are also available on Github ( https://github.com/psychoinformatics-studyforrest-paper-ironyannotation).
