# Supplementary material for: Lies, irony, and contradiction — an annotation of semantic conflict in the movie "Forrest Gump"
Source: F1000Res. 2016 Sep 26;5:2375. [Version 1] doi: 10.12688/f1000research.9635.1 (PMC5133679; doi:10.12688/f1000research.9635.1)
Supplement: Peer review history

# Peer review: Referee response for version 1

**DOI:** 10.5256/f1000research.10382.r17893

**Published:** 2016-11-29

**Authors:** Stéphanie Caillies, Alexandre Obert

**Affiliations:** 1 C2S Laboratory (EA 6291), University of Reims Champagne-Ardenne, Reims, France

In this data note, the authors analyzed and reported semantic conflicts in the movie “Forrest Gump”. This data collection has been well conducted and is part of a larger project named the studyforrest project.

 An explanation in few lines of what the goal of the studyforrest project is and of the rationale behind this data note would be welcomed. The authors mentioned that this data collection was conducted “in order to evaluate whether the available brain imaging data can be readily used to study this aspect of cognition” but it is quite hard to follow this sentence without information regarding the goal of studyforrest project and without reading the recent publications of Hanke and Ibe. As a more minor suggestion, we think it could be useful to add some references justifying the cues used in this dataset (see Attardo’s *et al.*, 2003).

We have read this submission. We believe that we have an appropriate level of expertise to confirm that it is of an acceptable scientific standard.

## References

[1] AttardoS.EisterholdJ.HayJ.PoggiI.: Multimodal markers of irony and sarcasm. *Humor*.2003;16(2) :243-260.

# Peer review: Referee response for version 1

**DOI:** 10.5256/f1000research.10382.r16597

**Published:** 2016-10-21

**Authors:** Dylan Wagner

**Affiliations:** 1 Department of Psychology, Center for Cognitive and Brain Sciences, The Ohio State University, Colombus, OH, USA

In this data note the authors provide a new annotation of the motion picture Forrest Gump focusing specifically on moments of semantic conflict in the movie. This annotation is designed to further enrich prior annotations of the movie and the associated functional neuroimaging dataset of participants viewing Forrest Gump. The authors do a fine job of describing exactly how semantic conflict events were segmented and provide details on the inter-rater reliability of these annotations. Moreover, providing the source code for calculating descriptive statistics and general quality assurance is much appreciated.

 I have only minor recommendations:

 Perhaps it is beyond the scope of a data note, but it may help the reader if the authors could expatiate on the various annotation categories. Specifically, the introduction could be expanded to say a few words as to why semantic conflict is interesting, why these particular dimensions were the ones chosen and what exactly each means in lay terms. 

 Second, the authors suggest in their introduction that there are insufficient semantic conflict events in Forrest Gump to be truly useful. Although I appreciate the candor, it's my opinion that we should first see what creative uses people can make of this annotation and the associated imaging dataset before we get too sullen!

I have read this submission. I believe that I have an appropriate level of expertise to confirm that it is of an acceptable scientific standard.
